# Role of Transient Receptor Potential Canonical Channels in Heart Physiology and Pathophysiology

**DOI:** 10.3389/fcvm.2020.00024

**Published:** 2020-02-25

**Authors:** Hairuo Wen, Judith K. Gwathmey, Lai-Hua Xie

**Affiliations:** ^1^Beijing Key Laboratory, National Center for Safety Evaluation of Drugs, National Institutes for Food and Drug Control, Beijing, China; ^2^Department of Cell Biology and Molecular Medicine, Rutgers University-New Jersey Medical School, Newark, NJ, United States

**Keywords:** TRPC channel, store-operated calcium entry, oxidative stress, arrhythmia, myocardial hypertrophy, myocardial infarction, atrial fibrillation

## Abstract

Transient receptor potential canonical (TRPC) channels are involved in the regulation of cardiac function under (patho)physiological conditions and are closely associated with the pathogenesis of cardiac hypertrophy, arrhythmias, and myocardial infarction. Understanding the molecular mechanisms and the regulatory pathway/locus of TRPC channels in related heart diseases will provide potential new concepts for designing novel drugs targeting TRPC channels. We will present the properties and regulation of TRPC channels and their roles in the development of various forms of heart disease. This article provides a brief review on the role of TRPC channels in the regulation of myocardial function as well as how TRPC channels may serve as a therapeutic target in heart failure and cardiac arrhythmias including atrial fibrillation.

## Introduction

Transient receptor potential (TRP) channels are transmembrane non-selective cation channels. In 1969, for the first time, Cosens and Manning found that the electroretinogram of a visually impaired mutant fly only displayed a transient response to steady light compared to a sustained response in the wild type fly (1), hence the name *transient receptor potential*. Since the first *trp* gene was identified by Montell and Rubin (2), 28 Trp genes have been found in mammals so far, and they have been shown to play an indispensable role in the regulation of important physiological and pathological processes such as cell contraction, cell proliferation and differentiation as well as cell death (3). Most TRP channels are non-selective cation channels that allow transmembrane transport of monovalent and divalent cations such as Na^+^ and Ca^2+^. TRP channel activity is regulated by physical e.g., mechanical tension (i.e., stretch), temperature, osmotic pressure, as well as chemical stimuli such as pH, Ca^2+^, reactive oxygen species (ROS), and G-protein-coupled receptors (GPCRs) signaling. A large number of recent studies have found that classical transient receptor potential canonical (TRPC) channels are involved in the regulation of cardiac function and are closely related to the pathogenesis of cardiac hypertrophy, fibrosis, arrhythmias, and myocardial infarction. For example, a series of gene knock-out studies found that TRPC1, TRPC3, and TRPC6 are associated with cardiac hypertrophy (4–6) and that deletion of or impaired function of TRPC3 leads to cardiac conduction block (7, 8). In addition, mechanosensitive TRPC6 (in atrial endocardium cells) is associated with stretch-induced atrial arrhythmias (9). The expression and electrical function of TRPC3 and TRPC6 channels have also been detected in cardiac fibroblasts and plays a role in cardiac fibrosis resulting in fibrosis-associated heart diseases (10, 11).

In this short review article, we specifically review recent progress in studies involving TRPC channel regulation and the involvement of TRPC in cardiac function and clinical pathologies. TRPC channels may serve as a therapeutic target in cardiac hypertrophy, heart failure, and cardiac arrhythmias. In addition, we also provide information on newly reported small molecule TRPC channel modulators.

## TRPC Channel Structure and Distribution

**Figure 1:**
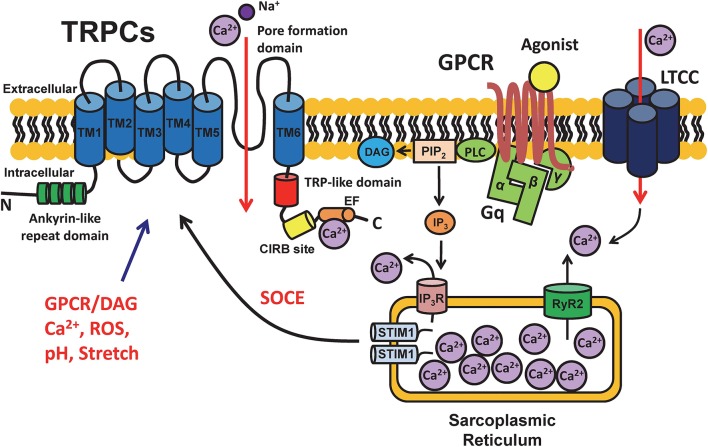
Schematic diagram of the structure of TRPC channels and cell signaling. TRPC channels are cation selective channels and have been demonstrated to have an intracellular amino terminus with ankyrin repeats (green) and carboxyl terminus with a TRP-like domain (red) for desensitization and a calmodulin- and inositol 1,4,5-trisphosphate receptor-binding (CIRB) site (yellow) for IP3 binding, as well as 6 transmembrane domains (blue) and a pore formation domain located between TM5 and TM6. TRPCs play important roles in mediating the intracellular Ca^2+^ signaling by incorporating with multiple functional proteins, e.g., G-protein coupled receptors (GPCRs), voltage-gated L-type calcium channels (LTCCs), ryanodine receptors (RyRs), and stromal interaction molecule 1 (STIM1). The schematic is a depiction of the general structure and regulation pathways of all TRPC channels. Structural and functional differences may exist between different TRPC subfamilies based on tissue type.

The TRPC channel is one of the most important members of the TRP family, which consists of six transmembrane domains (TMs) with the ion channel pore being located between the TM5 and TM6 domains [(12, 13); Figure 1]. TRPC (and other TRP) channels are not gated by voltage since they do not have a typical voltage sensor at TM4 (14). The amino terminus of TRPCs contains ankyrin repeats (12). There is a short phosphatidylinositol biphosphate (PIP_2_) regulatory region for channel activity and desensitization, which is known as the TRP-like domain for PIP2 regulation at the carboxyl terminus close to TM6 (15). In addition, a calmodulin/inositol 1,4,5-trisphosphate receptor-binding (CIRB) site and a Ca^2+^ binding EF hand are also located at the carboxyl terminus [(13, 16); Figure 1]. Seven different TRPC subtypes have been identified to date, i.e., TRPC1-7, which can form either homo- or heteromeric channels. Heteromeric channels formed by TRPC1/5, TRPC1/3, TRPC1/4, TPRC3/4, TPRC4/5 (15), and TRPC1/4/5 (17) have been described.

TRPC1, 3, 4, 5, 6, and 7 isoforms are widely expressed in a variety of human tissues and cell types (e.g., endothelial cells, fibroblasts, and muscle cells including cardiomyocytes) (18). It has been also determined that TRPC1, 3, 4, 5, 6, and 7 isoforms are expressed at the mRNA and protein levels in atria and ventricle. More specifically, TRPC1, TRPC3, TRPC4, and TRPC6 are found to be expressed in the sinoatrial (SA) node involving single pacemaker cells in mice (19). TRPC3 is the most dominate isoform in atrial and ventricular tissues, followed by TRPC1 and TRPC6 which contribute to around 10% of the level of TRPC3 (19, 20). TRPC3 and TRPC6 channels are found to be expressed in cardiac fibroblasts and mediate cardiac fibrosis (10, 11).

## Regulation of TPRC Channels and Cardiac Function

### TRPC Channels Are Involved in Myocardial Cell Signal Regulation

Intracellular Ca^2+^ is crucial in regulating various fundamental cellular processes. In cardiomyocytes, excitation-contraction (EC) coupling allows the heart to contract in a controlled manner. When the cardiac cell membrane is depolarized by an action potential, L-type Ca^2+^ channels (LTCC) are activated. This results in Ca^2+^ influx resulting in further Ca^2+^ release from the sarcoplasmic reticulum (SR) via type 2 ryanodine receptor (RyR2) in the form of a Ca^2+^ transient. Since the initial flow of Ca^2+^ into the cell (via LTCC) causes a larger release of Ca^2+^ within the cell from the SR, the process is called Ca^2+^ induced Ca^2+^ release (CICR). As a result, contractile myofilaments respond to the Ca^2+^ transient and this results in cardiac contraction.

In addition, a process of store-operated calcium entry (SOCE) has also been described in many cell types e.g., immune, neuron, and skeletal or cardiac muscle cells. Elevation in intracellular Ca^2+^ concentration can be modulated by sensing excessive release of Ca^2+^ from SR stores followed by the influx of extracellular Ca^2+^ through plasma membrane channels (21, 22). SOCE has been identified as a major process for increasing cytosolic Ca^2+^ load after SR Ca^2+^ depletion in many cell types (23). TRPC channels have been proposed to contribute to Ca^2+^ influx in SOCE (24). It has been shown that SOCE is significantly enhanced by the overexpression of TRPC channels while it is reduced by pharmacological and genetic knockdown of TRPC channels (25). TRPCs have been proposed to contribute to SOCE by forming a complex together with Ca^2+^ release-activated Ca^2+^ channel protein 1 (Orai1) and the Ca^2+^ sensor stromal interaction molecule 1 (STIM1) on the SR membrane (25–27). A mode of SOCE activation has been proposed by Birnbaumer et al. (28) in which STIM1 is activated subsequent to the depletion of SR Ca^2+^ stores and is promoted by the re-distribution of Orai and TRPC as well as the formation of a SOCE complex resulting in the regulation of Ca^2+^ influx. Importantly, SOCE has been reported in myocytes isolated from fetal, neonatal, and hypertrophic hearts (29, 30), however, its existence, molecular nature and pathological/physiological relevance in normal adult human hearts has not drawn much attention. Recent studies, including ours, have revealed TRPC channels play an important role in the regulation of electromechanical activity of the developing heart (8), Ca^2+^ paradox injury (31), pathological remodeling after myocardial infarction (32), as well as has a proarrhythmic effect under hyperactive conditions (by activators) (33). Hence, TRPC channels appear to be another important way by which Ca^2+^ can enter cardiomyocytes and regulate myocardial contractility in addition to voltage-gated Ca^2+^ channels (such as L-type or T-type) and the Na^+^/Ca^2+^ exchanger. In general, all TRPC channel subtypes are activated by GPCR and PLC activation and induces receptor operated Ca^2+^ entry (ROCE) that can function while Ca^2+^ store (SR/ER) is still filled (34). Interestingly, TRPC3, 6, and 7 are directly activated by diacylglycerol (DAG), while TRPC1, 4, and 5 are insensitive to DAG (35, 36). Furthermore, some TRPC channels (e.g., TRPC4 and 5) have been shown to be activated by intracellular Ca^2+^ (37–40), therefore presumably conferring a positive feedback loop (41) or functional interactions between TRPC and other Ca^2+^-permeable channels (e.g., LTCC) (38). It will need further studies to determine whether the effect of Ca^2+^ is direct or mediated by calmodulin.

### TRPC Channels Are Involved in Oxidative Stress-Induced Heart Disease

Overproduction of ROS could induce a variety of heart related illnesses including age related diastolic dysfunction, heart failure, ischemic injury, and arrhythmias. Compelling evidence has shown that hydrogen peroxide (H_2_O_2_) induces early after depolarizations (EADs) or delayed afterdepolarizations (DADs) as well as triggered activities (TAs) (42) in rabbit cardiomyocytes. Oxidation of Ca^2+^/calmodulin-dependent protein kinase II (CaMKII) and activation of late Na^+^ current and L-type Ca^2+^ current have been proposed to be the underlying mechanism(s). Additionally, an increasing number of studies have demonstrated that TRPC channels are the targets of oxidative stress regulation as well (43, 44). The upregulation of the expression of TRPCs is positively correlated with oxidative stress responses (43–45) suggesting that this regulatory pathway may play an important role in cardiomyocyte dysfunction resulting from increased Ca^2+^ overload. For instance, TRPCs are considered as important ROS sensors when heterogeneously expressed in endothelial cells and in the *mdx* mouse model of muscular dystrophy (46). H_2_O_2_ can possibly activate TRPC6 channels through the intracellular thiol group (47). Furthermore, TRPC3 has been shown to be involved in the up-regulation of ROS and CaMKII activities in mouse cardiomyocytes (45) while CaMKII inhibitors significantly reduce heterogeneously expressed TRPC6 channel activity *in vitro* (48). Another piece of evidence has demonstrated that NADPH oxidase 2 (Nox2) serves as a main source of ROS in cardiomyocytes and is able to form a stable complex with TRPC3 channels, while the TRPC3-Nox2 complex plays an important role in regulating doxorubicin-induced myocardial atrophy (49). In summary, a positive feedback loop mediated by TRPC-ROS-TRPC may play a significant role in the regulation of heart failure (50).

### TRPC Channel Modulators

**Figure 2:**
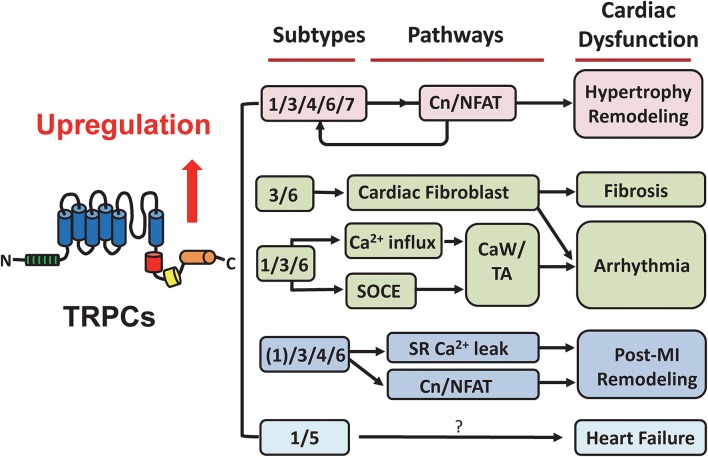
Summary of TRPC-associated cardiac dysfunction. TRPCs have been recognized as considerable mediators in the heart under pathological conditions. This suggests their potential in developing targeted drugs for cardiac diseases. The participation and underlying pathways of different TRPC subtypes in hypertrophic remodeling, fibrosis, arrhythmia, post-myocardial infarction remodeling, and heart failure are illustrated. See details in the main text. Cn/NFAT, calcineurin/nuclear factor of activated T cells; SOCE, store-operated calcium entry; CaW, calcium waves; TA, triggered activities; MI, myocardial infarction.

As previous mentioned, various knock out or transgenic overexpression mouse models have been used in studying the roles of TRPCs (4–6, 51). These genetic mouse models have been shown to be very useful for obtaining functional and pathological information as summarized in Figure 2. However, caution should be taken when explaining the phenotypic data, since many studies have been done using conventional knockout mice (46, 52). Establishing an inducible conditional TRPC knockout mouse model is needed in future studies.

Besides genetic approaches, pharmacological TRPC modulators have been widely used as a tool to define the functional roles of TRPCs. For example, Gd^3+^, 2-aminoethoxydiphenyl borate (2-APB), and SKF 96365 are generally used as non-selective TRPC blockers (8, 33, 52, 53). Some relatively selective blockers include Ethyl-1-(4-(2,3,3-trichloroacrylamide)phenyl)-5-(trifluoromethyl)-1H- pyrazole-4-carboxylate (Pyr3, a TRPC3 blocker) (54), GsMTx4 (a TRPC1 and TRPC6 blocker) (55). Intriguingly, recent studies have reported several selective small-molecule inhibitors for TRPCs. Pico145 and HC7090 selectively inhibit TPRC1/4/5 complex channels, while ML204 selectively blocks the TRPC4 channel (less selective to TRPC5) (17, 53). Another small molecule AC1903 has been identified as being more selective for TRPC5 channels (56). Seo et al. (6) has tested selective TRPC3/6 antagonists (GSK2332255B and GSK2833503A) and found combined TRPC3 and TRPC6 blockage generates similar anti-hypertrophic effects as those obtained with combined deletion of the TRPC3 and TRPC6 genes. On the other hand, some useful agonists/activators are also available for TRPC channel studies. For example, (–) Englerin A is an agonist for TRPC5 and TRPC4 (17). Hyperforin has been used as an agonist for TRPC6 (33). The identification of these new selective TRPC modulators provides possibilities for pharmacological therapy for TRPC-related diseases.

## TRPC Channels Are Potential Targets for the Treatment of Heart Disease

### TPRC Channels and Myocardial Hypertrophy

The heart function of TRPC-deficient mice showed no abnormality under normal conditions compared with wild type mice (46) suggesting that TRPC channels are not a key factor in maintaining normal physiological function of the heart at baseline. However, studies have shown that many TPRC channel subtypes play an important role in pathological conditions. The expression levels of TRPC1, TRPC3, and TRPC7 are up-regulated by angiotensin II (AngII), endothelin-1 (ET-1) and phenylephrine (57). TRPC3 and TRPC6 regulate a Ca^2+^ signaling pathway mediated by diacylglycerol (DAG), which is crucial in AngII-induced nuclear factor of activated T cells (NFAT) and cardiac hypertrophy (58). SOCE levels in transgenic mice expressing dominant-negative TRPC3, TRPC4, and TPRC6 channels are significantly lower than those in wild-type mice with transverse aortic constriction induced cardiac hypertrophy (46). In addition, the Ca^2+^ influx level and expression of hypertrophic markers in TRPC-deficient mice subjected to transverse aortic constriction were significantly lower than those in WT mice (51) suggesting that TRPC channels are potential therapeutic targets as an important endogenous regulator of pathological cardiac hypertrophy.

TRPC channels promote cardiac hypertrophy mainly by activating the calcineurin/nuclear factor of activated T cells (Cn/NFAT) pathway (46). Under the action of stimulating factors such as channel activators or myocardial stretch, TRPC channels might mediate Ca^2+^ influx and activate calcineurin leading to the translocation of NFAT and the activation of a myocardial hypertrophy cascade. Activation of the Cn/NFAT pathway could further up-regulate various TRPC channel subunits such as TRPC1, TRPC3, TRPC4, and TRPC6 resulting in sustained hypertrophic remodeling (46, 51, 57). It is notable that, in contract to hypertrophy, a significant increased expression of TRPC5, and to a less extent, TRPC1 was observed in failing human heart samples (4, 59).

### TPRC Channels and Cardiac Arrhythmias Including Atrial Fibrillation

Atrial fibrillation (AF) is the most common persistent arrhythmia. It has been suggested that TRPC channels can affect the development of AF by regulating the function of cardiac fibroblasts (11) It was found that the inhibition of TRPC3 expression can reduce the AngII-induced Ca^2+^ influx and extracellular signal-regulated kinase (ERK) phosphorylation thereby reducing the proliferation of fibroblasts (60). The expression of TRPC3 in the atrium is elevated in patients with AF. Therefore, decreasing TPRC3 channel-mediated Ca^2+^ signaling could reduce susceptibility to the development of AF (61). The important role of TRPC3 in AF generation was further demonstrated by Ju et al. who showed that pacing-induced AF in angiotensin II-treated mice are significantly reduced in TRPC3 knockout (TRPC3^−/−^) mice (62). Additionally, it has also been shown that activation of TRPC3 by adenosine receptor stimulation may disturb atrioventricular conduction (7). Fibroblasts of TRPC6 knockout mice failed to differentiate into normal myofibroblasts under the action of TGF-β1 and had an associated lower cardiac function with a higher mortality rate after myocardial infraction (MI) (63). Hence, the TRPC channels may serve as potential therapeutic targets for AF associated with fibrotic lesions.

In addition, TRPCs are directly associated with electrical signaling. *Ex vivo* electrocardiograms demonstrated that in the presence of TRPC channel inhibitors SKF-96365 and Pyr3 resulted in PR and QT interval prolongation, first and second degree atrioventricular block as well as a reduction in intra-ventricular conduction in the developing heart (8). Since TRPC1 and TRPC6 belong to stretch-activated channels (SACs), acute elevation of atrial pressure can promotes AF while the TRPC (also a stretch-sensitive channel) blocker GxMTx4 effectively inhibits the occurrence of AF (64, 65).

As for in the ventricle, our group, and others have recently determined that SOCE, which is at least partially mediated by TRPC channels, exists in adult mouse ventricular myocytes (31, 33). TRPC channels and SOCE may be involved in cardiac arrhythmogenesis via promotion of spontaneous Ca^2+^ waves and triggered activities under hyperactivated conditions (33). These data also suggest an underlying mechanism for one of the side effects of St. John's Wort (hyperforin a TRPC6 activator) i.e., heart palpitations. Therefore, caution is required when treating depression in patients who also have heart disease and in particular arrhythmias.

### TPRC Channels and Myocardial Ischemia/Infarction

In the mouse myocardial infarction (MI) model, it has been shown that mRNA expression levels of TRPC1, TRPC3, TRPC4, and TRPC6 subtypes is significantly increased from 1 to 6 weeks after MI (32). This study revealed that TRPC channels induce SR Ca^2+^ leakage and interfere with normal Ca^2+^ levels in excitation-contraction coupling microdomains thereby reducing myocardial contractility subsequent to ischemic conditions. This effect can possibly be attributed to the Ca^2+^-activated Cn/NFAT signaling pathway via caveolae membrane microdomains. Blocking TRPC channel activity after 1 week post-myocardial infarction significantly improved myocardial structure and function (32). The brain-derived neurotrophic factor (BDNF) increased significantly after 3 days post-MI, significantly reduced infarct size, decreased serum lactate dehydrogenase activity, and played a protective role in myocardial infarction. Hang et al. (66) found that TRPC3/TRPC6 is required for a BDNF-mediated inhibition of apoptosis in cardiomyocytes and protected the myocardium during myocardial hypoxia-ischemic injury (66). In addition, Satoh et al. found that TRPC7 channels mediate cardiomyocyte apoptosis through the regulation of intracellular Ca^2+^ levels by inducing a Ca^2+^-dependent kinase-regulated pathway (67). Taken together, these results suggest that TRPC channels may represent new therapeutic targets and their blockade might effectively alleviate pathological remodeling of myocardial structure and function thereby maintaining contractile reserve after MI.

## Conclusions

TRPC channels as important cell sensors in the human body have drawn extensive attention in the fields of nervous system diseases and glomerular diseases. However, their roles in the induction and development of heart disease are still unclear. Different TRPC subtypes are widely expressed in the human heart including SA node, atrium and ventricle (19, 20). Under pathological conditions, TRPC channel mediated Ca^2+^ signaling may be closely related to the development of various cardiac pathologies such as cardiac hypertrophy, fibrosis, cardiac arrhythmia, and post-MI remodeling via mechanisms involving ROS and NFAT (Figure 2). In recent years, small molecule selective antagonists against TRPC3 and TRPC6 have been developed, such as GSK417651A, GSK2293017A, GSK2332255B, GSK2833503A etc. (6, 16). These compounds have been used in experimental research laboratories to study the function of TRPC3 and TRPC6 channels but have not yet officially entered the clinical trial stage. A thorough understanding of the molecular mechanism of TRPC channel-induced pathological conditions and the regulatory pathways/sites involved in key sites contributing to the regulation of myocardial function will undoubtedly be of great help in the design of therapeutic drugs targeting TRPC channels.

## Author Contributions

HW, JG, and L-HX reviewed literatures and wrote the paper. HW and L-HX prepared the figures.

### Conflict of Interest

The authors declare that the research was conducted in the absence of any commercial or financial relationships that could be construed as a potential conflict of interest.
